# History of Rabies Incidence and Rabies Control in Serbia in Support of the Zero by 2030 Campaign to Eliminate Dog-Mediated Human Rabies

**DOI:** 10.3390/v14010075

**Published:** 2021-12-31

**Authors:** Srđan Stankov, Dušan Lalošević, Anthony R. Fooks

**Affiliations:** 1 Department of Microbiology, Pasteur Institute Novi Sad, Hajduk Veljkova 1, 21000 Novi Sad, Serbia; dusan.lalosevic@mf.uns.ac.rs; 2 One Health Association of Serbia, Hadži Ruvimova 23, 21000 Novi Sad, Serbia; 3 Faculty of Medicine, University of Novi Sad, Hajduk Veljkova 3, 21000 Novi Sad, Serbia; 4 Animal and Plant Health Agency (APHA), OIE Reference Laboratory for Rabies/WHO Collaborating Centre Communicable Disease Surveillance and Response Collaborating Centre for Rabies, Weybridge KT15 3NB, UK; Tony.Fooks@apha.gov.uk

**Keywords:** Serbia, rabies, prophylaxis, diagnosis, surveillance, vaccination

## Abstract

Urban (principally canine-mediated) rabies has been a public health risk for people living in Serbia for centuries. The first legal act in urban rabies prevention in Serbia was established in 1834 by introducing high taxes for pet dog owners. Five years later in 1839, the first set of literature describing rabies prevention was issued by the health department from The Serbian Ministry of Interior. An overview of cauterization of rabies wounds was presented as the principal method of rabies post exposure prophylaxis. In 1890, a human rabies vaccination was introduced in Serbia with the royal government directive which ordered patients to be treated at the Pasteur Institute in Budapest in receipt of rabies vaccination. Urban (canine) rabies was eliminated during the 1980s, but sylvatic (principally fox-mediated) rabies still prevailed. The last human rabies case was recorded in the Province of Kosovo and Metohija in 1980. Sylvatic rabies in Serbia is in the final stages of elimination by orally vaccinating foxes (*Vulpes vulpes*). The only published finding of a lyssavirus among Serbian bats was made in 1954 by Dr Milan Nikolić in the vicinity of Novi Sad. In 2006, a comprehensive two-year active surveillance program of lyssaviruses in bats in Serbia was undertaken. In this single study, all of the bats from Serbia tested negative for a lyssavirus.

## 1. Introduction

The history of diagnostics, the control of rabies, and human rabies prophylaxis in Serbia has been challenging, largely reflecting the turbulent history of ethnicities and states in this and the wider European area. Throughout history, the territory of today’s Serbia has been the scene of mass migrations of people and mutual struggles of ethnic groups. Under such conditions, written records of lifestyle habits, social customs, and activities of public importance were not always preserved. Historically, the Austro-Hungarian and Turkish empires were the main political forces that decisively influenced life in Serbia. The Austro-Hungarian authorities made a substantial and crucial effort to implement public health measures in support of the control of infectious diseases in Serbia. With regard to the epidemiology of rabies, considered one of the most dangerous and severe infectious diseases in Serbia, typical dog (*Canis lupus familiaris*) husbandry practices differed significantly throughout the country. In Austria, the service for catching and removing stray dogs had been established and was effectively functioning. In contrast, the presence and movement of owned dogs in populated areas of Turkey was tolerated without owner supervision, thus enabling the maintenance and growth of an unowned dog population, creating a major naïve population of dogs and thereby sustaining the spread of urban rabies [1]. These were the main historical events that shaped both the social and natural factors determining rabies incidence and opportunities for rabies control in Serbian territory at a time preceding the formation of the Serbian state.

The purpose of this historical review is to expose the dynamics of rabies appearance in Serbia in humans and domestic and wild animals and the development of methodology for rabies control, with special emphasis on the mutual interaction of disease appearance and specific control measures. This historical review should be useful for future public health policies for the control of emerging infectious diseases from a One Health perspective, especially in low- and middle-income countries.

## 2. Historical Review of Incidence and Control of Rabies in Serbia from Its Constitution as an Independent Country in the 19th Century

### 2.1. Incidence of Rabies in Serbia in the 19th Century

From the earliest records on the emergence and control of rabies in Serbia, a historian of veterinary and human medicine, Dragoljub Divljanović, cites the report of Dr Jovan Mašin from 1848, who reported a case of dog rabies. The dog bit two children, and the therapy that the doctor applied was to cauterize the wounds well, followed by a recommendation of a strict diet and powder from Spanish beetles “pulvis cantharidum” [2]. The obvious frustration of medical doctors and lack of efficacious medical interventions in treating rabies in the Serbian medical literature was vividly reported by the doctor and writer Laza Lazarević with the description of a case of rabies in a twelve-year-old girl. “In addition to our helpless therapy, which consisted of chloroforming exclusively due to the child’s absolute impossibility of swallowing (we meant to at least alleviate the attacks), eclampsia rapidly took hold and the child died at two o’clock in the morning, around 16 h after the first symptoms.” [3]. It is not surprising that at the time of formation of the Principality of Serbia at the beginning of the 19th century, rabies control was one of the priorities of public health, which was then managed by the Sanitary Department at the Serbian Ministry of Interior.

### 2.2. Legal Acts and Measures for Dog Rabies Control

The first legal act aimed at the prevention of urban rabies, established in 1834, was the introduction of high taxes for dogs kept only as pets in order to limit the number of such dogs. However, taxes were exempt from dog owners who used dogs as herd keepers of livestock. Five years later, in 1839, the first collection of professional publications on rabies prevention in Serbia was issued, entitled “Lessons on Rabies to All Chiefs and Trustees of Justice” published by the Sanitary Department of Ministry of the Interior and then distributed to local authorities and the public. In this guide, although the etiology of rabies was misunderstood, a precise description of the clinical picture of canine rabies was provided. In addition, many methods of treating rabies recommended by traditional medicine and folk customs were declared as inappropriate and ineffective, despite the relatively poor knowledge of facts and details about the onset, pathogenesis, and prophylaxis of rabies. The most important measure of post-exposure prophylaxis (PEP) of rabies in humans recommended the thorough cauterization of the wound itself after a bite from a ’suspect’ rabies virus-infected dog. In 1858, a new public document was issued entitled “Public Orders Against Rabies”, which ordered the culling of specific animal species considered as reservoirs capable of transmission of rabies virus, especially wolves (*Canis lupus*). Further, killing of rabid or rabies-suspected animals was ordered, along with the confinement of animals that have been in contact with these animals, followed by their observation for four weeks. These measures were accompanied with instructions for disinfection and safe disposal of infected animal carcasses. In 1880, the Belgrade District Court banned the release of dogs in public areas without supervision of the owners and obliged the owners to put a muzzle on dogs while walking on a public place. In addition, a special sanitary service for catching stray dogs was established which placed these dogs in quarantine. The service gave the dog owner a period of 48 h to report to quarantine; otherwise the dog would be euthanized.

In 1881, a general legal act for the control of infectious diseases in animal husbandry was issued entitled: “Law on Protection Against Livestock Infections and Measures for Control of These Infections”. With regard to rabies prevention, this law described the procedures of quarantine, surveillance and procedures for humane destruction of rabies ‘suspect’ animals, especially those that have previously injured a human or another animal. Following instruction by the Minister of the Interior in 1881 on the implementation of this law under Article 34 on rabies of domestic animals, paragraph 2 states that in case people or animals are bitten by a rabies ‘suspect’ animal, such an animal will be safely caught and detained. It must not have been killed immediately, but only after an expert examination confirmed whether the animal was clinically rabid or not. By order of the police authorities, such animals were detained for further observation. Any animals destroyed during quarantine or death during this observation period were further investigated. Two years after adoption of this legal act, a comprehensive and permanent record of domestic animals suffering from rabies in Serbia was established, with rabies cases being diagnosed according to clinical signs and supported by macroscopic pathological findings (Table 1).

Direct records of rabies in wild animals were not kept at that time but are based on the data on post-exposure prophylaxis of patients in the Pasteur Institute in Niš between 1901 and 1904. Interestingly, sylvatic rabies was not considered a major public health problem at that time. Unfortunately, the presence of urban rabies in Serbia at the end of the 19th century was associated with a substantial number of rabies cases in humans, primarily due to unsystematic and incomplete application of rabies control measures in dogs [2].

## 3. Establishment of the First Serbian Pasteur Institute in Niš

**Figure 1:**
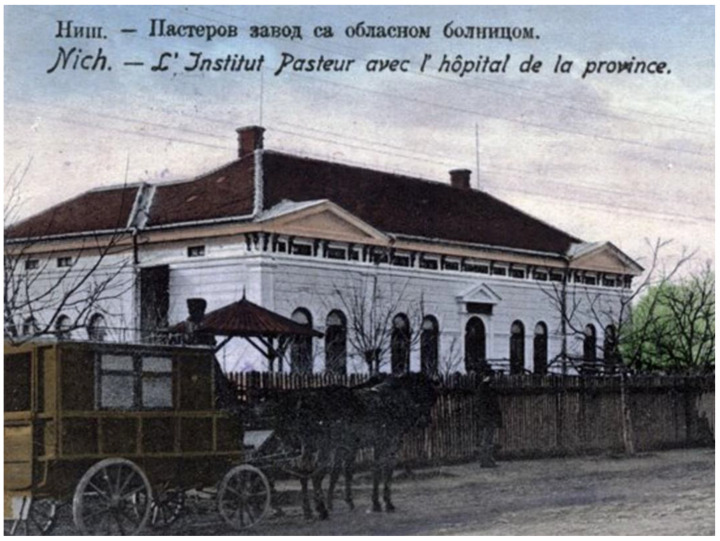
Pasteur Institute in Niš (picture made about 1910, originating from archive collection of Museum of Health Culture in Niš, and taken from https://sr.wikipedia.org/sr-ec/Пастеров_завод_у_Нишу, accessed on 23 December 2021).

The victory of Pasteur’s science and vaccination against rabies had important repercussions throughout Serbia. Less than a year after vaccination of Pasteur’s first patient, King Milan decorated Pasteur with the highest order of St. Sava. In 1886, Serbian newspapers reported the work of Louis Pasteur in the article “Rabies in Europe and in the World”. The text “On Canine Rabies—Diagnosis and Prophylaxis” by veterinarian Antonio Kobliška was published in 1896 by the Serbian Archives of Medicine [4]. Practically, human rabies immunoprophylaxis in Serbia began in 1890 with the order of The Royal Government by instructing that injured patients should be sent for vaccination to Budapest [5]. King Milan soon approved the establishment of the first Pasteur Institute in Serbia, which began operating in Niš in 1900 (Figure 1). In the first years upon its establishment, this institute dealt not only with rabies prevention but also with vaccination against smallpox. For rabies PEP, patients were then treated using the Hegyes dilution method, which was just as effective as Pasteur’s original drying method in preparing the vaccine [6]. Between 1901 and 1910, only 34 cases out of a total of 3825 patients subjected to rabies PEP had an unfavorable outcome (i.e., rabies death), which represented an immunization failure of only 0.89%. [7]. In the same period, the most injured patients were from dog bites (89.6%), followed by cats (4.6%) and other domestic animals (4.3%). There were only nine reported injured patients from wolves (0.8%). By 1915, the number of those treated increased, and a total of 8649 people had been treated [5].

Surveillance of the population of dogs and other domestic animals in the period immediately after the First World War was considerably weakened, so there was a sudden expansion of unowned and stray dogs in populated areas. As a result, the number of people injured by dogs and the number of rabies PEPs increased significantly. For example, in Niš in 1921, the number of treated patients was 2022. In the same year, 1837 patients were taken care of in the Zagreb institution, and another 1083 people in the department of this institution in Velika Gorica [8]. Immediately after the end of the First World War, the Pasteur Institute in Niš was reconstructed under the management of Dr Gerasim Alivisatos, a new director originally coming from Greece. Between 1919 and 1920, an increased percentage of unsuccessful rabies PEPs was observed and, consequently, there was a need to improve the vaccination scheme as well as the quality of the rabies vaccine itself. To that end, Dr Alivisatos improved the quality of the vaccine by introducing the procedure of attenuation of the vaccine virus using ether, the method of ‘etherization’ [9]. In that way, it was possible to inject a much larger amount of vaccine to the patient on the first day of PEP. The administration of this new vaccine to humans was preceded by an extensive experimentation in sheep, in which the safety of the new PEP was first examined by giving each sheep 44 g of experimental vaccine in the abdomen for 66 days. The health of the experimental animals was monitored for 18 months without any signs of clinical disease demonstrating that the prototype vaccine was generally safe. A new procedure for immunizing injured people, called the “Alivisatos ether method”, included the application of a total of 10 g of brain suspension with rabies virus previously inactivated with ether, for 13 to 15 days during the entire procedure. Initially, the effects of the new method were monitored in parallel with the results of the application of the previous Hegyes dilution method. The effectiveness of the Alivisatos method was reported in a group of 315 patients, with the most severe injuries, who all survived more than one year after PEP. In contrast, 11 from a total of 287 treated patients fell ill and died from the group with alternative routinely applied PEP scheme within 15 days after the procedure. The complete absence of any signs of damage to the nervous system treated by the new method in a group of over 1100 patients who were continuously monitored supported the importance of this new approach. Due to these very favorable results, the new method of immunization against rabies according to Alivisatos was soon adopted as a routine procedure not only in all Yugoslav Pasteur institutes, but also in Pasteur institutes in Athens, Vienna, Sofia, Madrid and Buenos Aires. The importance of introducing the Alivisatos ether method was indicated primarily by the fact that the number of post-exposure treated patients in the Kingdom of Serbs, Croats and Slovenes (SCS) between 1921 and 1925 was among the highest in Europe, with an annual average of over 5500 cases, of which a third were treated in Niš [10,11]. At the same time, in other areas of the Kingdom of SCS, a notable increase in the number of treated patients was recorded, in Vojvodina from 610 in 1921 to 1170 in 1925; in Central Serbia from 414 in 1924 to 558 in 1925; and in Montenegro from zero cases in 1921 to 157 in 1925, all counted per 100,000 inhabitants of the defined areas. A total of 27,906 people were treated with PEP in the Kingdom of SCS between 1921 and 1925, of which rabies was registered in 93 of human cases despite PEP. Of these, 38 cases were registered in Central Serbia and 13 in Vojvodina. The incidence of human rabies cases showed periodicity every two to three years, with a minimum of five to nine cases and a maximum of 22 to 27 cases per year. This periodicity was most likely conditioned by the periodic occurrence of this disease in animals, for which there were not any direct data. In addition, according to many doctors who dealt with rabies PEP, including Alivisatos himself, measures to combat rabies in animals were not implemented during this period. The first recorded sanitary measure against urban rabies in the Kingdom of SCS was undertaken in 1925 and 1926, when the population of unowned and stray dogs and cats was reduced by culling. In the period between 1919 and 1928, a total of 12,856 patients were treated against rabies at the Pasteur Institute in Niš, with the vaccination failure rate being 0.33% [12].

## 4. Establishment of the Pasteur Institute in Novi Sad

**Figure 2:**
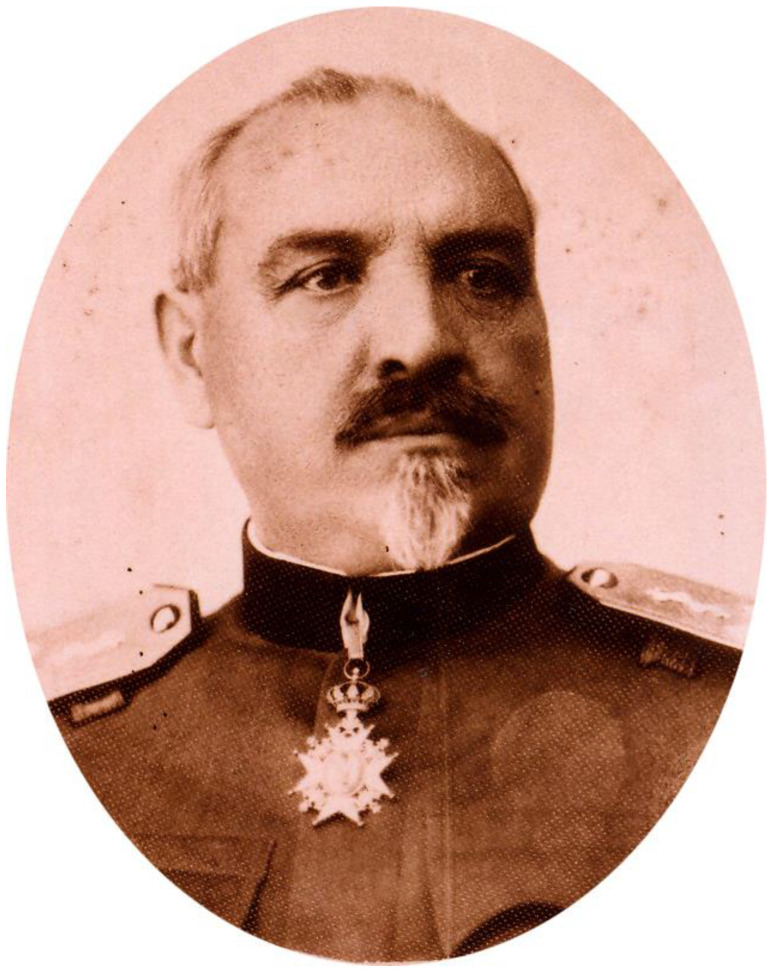
Dr Adolph Hempt (by courtesy of Mrs Dagmar Hempt-Mirić (1913–2013), the daughter of Dr Adolph Hempt).

In 1921, the second Pasteur Institute in Serbia was founded in Novi Sad to provide rabies protection services on the territory of northern Serbia, including Belgrade, where the highest incidence of this most dangerous zoonosis was recorded at that time. Dr Adolf Hempt, an Austro-Hungarian military physician and later a municipal doctor, born in Novi Sad in 1874, was appointed the first director of the Pasteur Institute in Novi Sad (Figure 2).

Since its establishment, the services of the new institution have been used not only by patients from the northern part of Serbia, but also from eastern Croatia and northern Bosnia, so that the total number of patients was large. Dr Hempt used the original Hegyes method until in 1922, he became acquainted with the Alivisatos ether method, which was then adopted as the only method in routine use in the Novi Sad institute. Initially, out of a total of 234 patients, only 1 patient died of rabies (PEP failure rate 0.42%). According to available publications, the Hempt vaccine was the first in Europe and second after the Semple vaccine developed as a completely inactivated rabies vaccine, unlike the Alivisatos vaccine which apparently contained a minute amount of live virus. This allowed Hempt to increase the doses of vaccine while shortening the PEP period. For very severe injuries, Hempt shortened the duration of PEP to only five days, calling this modification the “fast ether method” or the “Serbian method”. By 1933, a total of 6368 people were treated with this method, with only 5 cases of failure (0.08%) and 8 cases of neurological complications (0.13%) [5]. By the end of 1927, Hempt had succeeded in developing a procedure for preparing a vaccine with phenol as a preservative, which enabled the vaccine to be stored for at least one year, as well as to transport the vaccine over long distances. Such a vaccine became known as the “ether-phenolic vaccine” or the “Hempt vaccine” [13]. This put an end to the need for decentralized preparation of the rabies vaccine, and soon all other Pasteur institutes in the country were closed except the Novi Sad institute, which produced the vaccine for the needs of entire country [14]. Rabies prophylaxis of people in Serbia was then undertaken until the end of the 1970s by applying the Hempt vaccine according to the stated fast ether method. The vaccine was produced at the Pasteur Institute in Novi Sad and then distributed to over 100 other anti-rabies stations, where it was administered to patients previously injured by animals. Despite a very effective PEP of human rabies, the number of human cases of rabies at the time of urban rabies was closely correlated with the number of cases in animals. Since the early 1980s, Hempt vaccine was gradually replaced by modern inactivated rabies vaccines produced using cell culture techniques. Somewhat earlier, a procedure of passive rabies immunoprophylaxis was introduced, since the production of equine rabies immunoglobulin (ERIG) began in 1970 at the Institute of Immunobiology and Virology Torlak in Belgrade. After that, imported human rabies immunoglobulin (HRIG) was introduced in parallel as the most advanced and safest preparation. Continuous production of HRIG in Serbia began in 1991 as a result of cooperation between the Blood Transfusion Institute of Serbia and the Pasteur Institute in Novi Sad, which eliminated the need for both ERIG and imported HRIG. The last human rabies cases were recorded in 1964 in Vojvodina, in 1976 in Central Serbia, and in 1980 in Kosovo [15].

## 5. Eliminating Canine-Mediated Urban Rabies

Data on animal rabies in the first half of 20th century were scarce and irregular, but it was obvious that canine rabies was the most serious problem. According to Vuković, in 1931 cases of animal rabies were recorded in 376 dogs, 19 cats, 33 cattle, 15 pigs, and 10 horses, while in 1932 rabies positive were 619 dogs, 36 cats, 45 cattle, 27 pigs, 12 horses, and 4 goats. At the same time, the number of affected municipalities increased from 285 in 1931 to 447 in 1932 [16]. Measures to control and eliminate canine rabies began in the mid-1920s. In addition to removing stray dogs, many veterinarians advocated for vaccinating animals, primarily dogs against rabies. From 1926 to 1933, the basic strategy of animal rabies prophylaxis was post-exposure vaccination with two doses of inactivated ether-phenol vaccine and the third dose of the so-called Gonsalves lipovaccine [13]. This regimen was then replaced with three doses of ether-phenol vaccine for three consecutive days. By 1935, a total of 2910 domestic animals that underwent this prophylaxis had a mortality rate of only 0.68%, while none of the 190 vaccinated dogs became ill [13]. During and immediately after the WWII, the incidence of canine rabies remained high, so that dogs accounted for over 50% of all cases of rabies in animals.

In Yugoslavia between 1946 and 1957, there were 5489 (56%) rabid dogs from a total of 9785 rabid animals, and urban dog rabies remained the dominant form of rabies epizootic until the 1980s. The prophylaxis of canine rabies in that period was supported by using pre-exposure vaccination of owned dogs according to Hempt, organized by campaigns started in 1947, along with the regular capture and culling of stray dogs. Urban rabies was reduced to sporadic dog rabies cases by 1981, which coincided with the elimination of human rabies cases, the last of which was recorded in 1980 in Kosovo province [15]. In 1981, the Hempt vaccine was replaced by the live attenuated Flury HEP vaccine from chicken embryos [17], which lasted until late 1990s, when an inactivated rabies vaccine prepared on a baby hamster cell culture (BHK) was introduced. Today, dog-mediated rabies is controlled by appropriate pet travel regulations harmonized with corresponding EU legislation [18]. Until the 1950s, rabies was diagnosed by the microscopic examination of Negri bodies as well as virus isolation in mice and rabbits. The method of isolation in rabbits was especially important for the differential diagnosis in cases of pseudorabies (i.e., Aujeszky’s disease).

At the Pasteur Institute in Novi Sad, the direct immunofluorescence technique (FAT) [19] was introduced in 1968, while the virus isolation test on mice (MIT) [20] had still been retained as a confirmatory method, even if this method is strongly encouraged to be replaced by faster and more ethical alternative techniques such as molecular tests or virus isolation on cell culture.

## 6. Emergence and Elimination of Sylvatic Rabies in Serbia

During the winter months in 1952–1953, rabies virus was transmitted to a fox population presumably from rabid wolves in Deliblatska peščara region and then spread autonomously among foxes (*Vulpes vulpes*). In this region many bovine rabies cases were also reported. This epizootic was independent from the established fox-mediated rabies epizootic from Poland, which was evident from its spatial isolation from other sylvatic rabies affected areas. After 10 years without sylvatic rabies, the second epizootic appeared in 1962 in the border region between Hungary, Romania, and Yugoslavia, but soon disappeared. During 1977, sylvatic rabies spread to Vojvodina Province, the northern part of Serbia, from the neighboring territories of Hungary and Romania, while moving south towards the Sava and Danube rivers. The fox rabies epizootic then slowed for many years, so that fox rabies in Central Serbia appeared later in 1986. Fox-mediated rabies then spread south in 1998 to Kosovo and Metohija. Apart from rivers as physical barriers, the relatively rare population of foxes as the main reservoir and vector of rabies virus infection in this area probably contributed to a slower spread of the epizootic [15,21]. Molecular studies of rabies viruses on the Balkan Peninsula revealed that a distinct group of Serbian fox rabies viruses provided further evidence for the southward wildlife-mediated movement of rabies from Hungary, Romania and Serbia into Bulgaria [22].

In 2010, a Veterinary Directorate as the national competent authority for animal health in Serbia initiated a long-term project of oral rabies vaccination of foxes and other wild carnivores, co-funded by the EU. Since 2011, the monitoring of the effectiveness of oral vaccination campaigns has been continuously conducted. These results showed that oral rabies vaccination of wildlife in Serbia was successful and characterized by a steady increase of vaccine bait uptake and immune response to the vaccine. As a result of these applied prophylactic measures, the number of reported rabies cases has steadily decreased until 2018, when the last positive rabies case in Serbia, in a fox in Krupanj municipality was confirmed [23]. The timeline of important achievements in rabies prophylaxis in Serbia is shown in Table 2.

## 7. Passive and Active Surveillance of Lyssaviruses in Bats in Serbia

**Figure 3:**
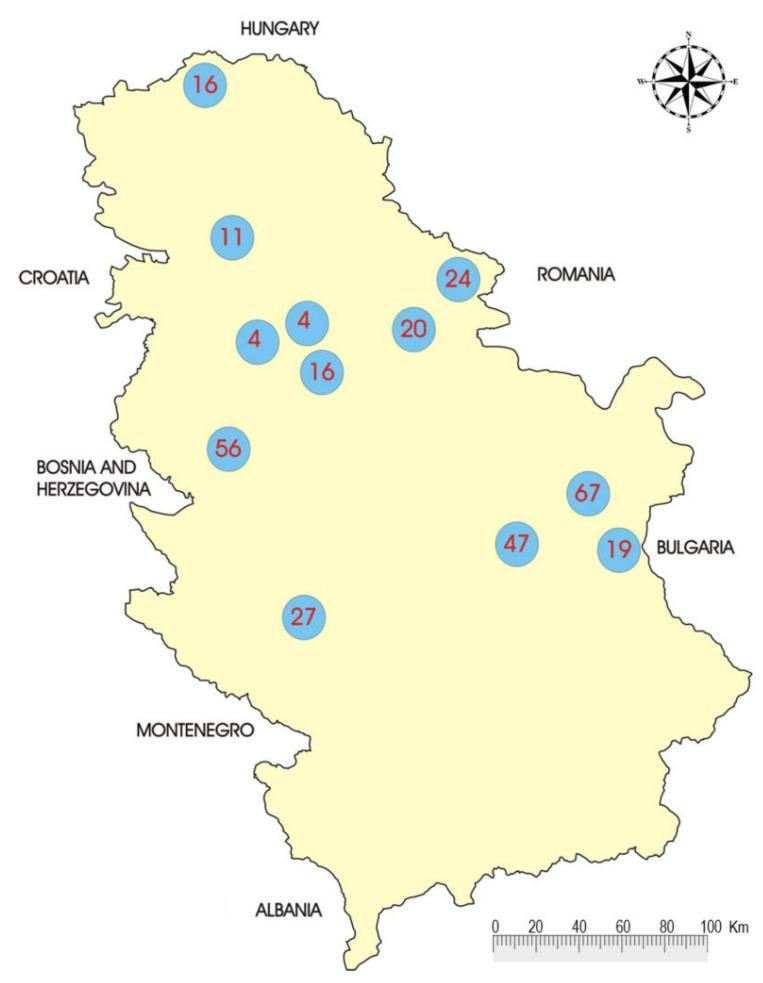
Locations and numbers of sampled bats in Serbia (2007–2008). Sampling locations are presented by circle positions on the map, and numbers of bats sampled at each location are shown in corresponding circles (from [25]).

Independently of routine surveillance of rabies in terrestrial animals, which was most often associated with the danger of direct transmission of rabies virus to humans, an active program, albeit limited in scope, of examination of rabies in insectivorous bats was established in 1955 and undertaken by Nikolić and Jelesić [24]. They isolated a virus from bats from the species common noctule bat (*Nyctalus noctula*) collected at the Petrovaradin Fortress, which, according to serological tests gave a positive reaction, suggesting a strain of rabies virus. There were little chances that the sample was false positive since it was immediately identified as rabies virus with neutralization by a specific anti-rabies serum, and the only practical possibility for a false finding was eventual sample mishandling. This isolate was then intensively passaged in mice and rabbits, with determination of its pathogenic properties and histopathological lesions. Research on lyssaviruses in bats continued the following year in the vicinity of Čortanovci, Petrovaradin and Novi Sad, but all with negative results. Further examinations of rabies lyssaviruses in bats continued between 1996 and 1997 when 37 specimens of 14 bat species were tested at The Pasteur Institute Novi Sad in collaboration with experts from The Natural History Museum in Belgrade. Rabies virus was not detected in any of these specimens by either FAT or by the MIT methods [25]. In 2006, The Veterinary Directorate of Ministry of Agriculture, Forestry and Water Management of the Republic of Serbia granted the project of two-year active surveillance of lyssaviruses in bats in Serbia. Enzootic fox rabies was still present in the territory (on average 192 laboratory confirmed rabies cases annually in the last 4 years) with sporadic spillover events into domestic animals. A total of 311 bats were sampled using mist netting sessions in 14 roosts throughout the country (Figure 3). Each sampled bat was identified and measured for the body parameters; saliva and blood were taken, and after rehydration and banding the bats were released at the same place where sampling took place.

Out of 20 sampled bat species in total (Table 3), the most frequent bats were Daubentons bats (*Myotis daubentonii)* (*n* = 44), lesser mouse-eared bat (*Myotis blythii*) (*n* = 41) and the Mouse-eared bat (*Myotis myotis*) (*n* = 33).

Sera from 184 bats were collected and tested at the Pasteur Institute Novi Sad with a modified RFFIT [26] using European bat lyssavirus 1 (EBLV-1) and European bat lyssavirus 2 (EBLV-2) obtained from The Animal and Plant Health Agency, UK. The presence of specific anti-EBLV-1 and EBLV-2 neutralizing antibodies was not detected in any of the tested samples. The presence of viable and infective European bat lyssaviruses in 271 saliva samples was tested by MIT [20] at the Pasteur Institute Novi Sad, and all test results were negative. Saliva samples, 232 in total, were also tested for the presence of lyssavirus RNA by RT-PCR [27] at the Institute of Veterinary Medicine of Serbia in Belgrade and were found negative. Additionally, a total of 82 bats previously sampled (2002–2008) by bat biologists from The Natural History Museum in Belgrade were included in this research. All brain samples were tested by FAT [19]. There was not any antigen in any of the samples tested [25]. The project of bat lyssavirus surveillance in Serbia did not show any indication for the presence of lyssaviruses in any of the bats tested. However, since the serological testing was only undertaken against EBLV-1 and 2, other phylogroup I and III lyssaviruses found in European bats [28] should also be investigated in future studies, since protective rabies immunity in vaccinated humans seems to be principally determined by lyssavirus phylogroup matching of vaccinal and challenge lyssavirus strains [29,30]. Based on this single bat rabies survey, there is still not any conclusive evidence that bat rabies is absent from Serbia, and consequently human exposures to bats in Serbia always require PEP. An important question to which we do not have a clear understanding is why more rabid bats have not been detected in Serbia since the first case in 1954. One possible explanation is that the bat population in Serbia was drastically reduced after 1950-ties so that the transmission of bat viruses was interrupted. Indeed, bats became protected species in Serbia since they had become an endangered species throughout Europe. Another probable explanation is that existing bat lyssaviruses remained undetected in recent surveillance studies as the numbers of bats tested had not been optimized for the detection of a bat lyssavirus.

## 8. Conclusions

In Serbia, canine-mediated human rabies has been eliminated, with the last human case of rabies (following a dog bite) being reported in 1980. Conversely, fox-mediated rabies still occurs and is in the final stages of elimination by using the method of oral vaccination of susceptible wildlife species. In a study of lyssaviruses in bats from Serbia, none of the bats captured tested positive for the presence of a lyssavirus. Serbia therefore remains compliant with the Zero by 2030 campaign and is in the final stages of the rabies elimination endgame.

## Figures and Tables

**Table 1:** The first systematic record of rabies in domestic animals in Serbia (1883 to 1914) (from [2]).

Year	Dog	Cat	Cattle	Sheep	Pig	Horse	Total
1883	12	-	18	-	3	-	33
1884	27	-	29	-	-	-	56
1885	-	-	7	-	-	-	7
1886	1	-	-	-	-	1	2
1887		-	6	3	3	-	12
1888	1	-	5	-	-	1	7
1889	-	-	2	-	-	1	3
1890	3	-	5	-	1	-	9
1891	1	-	3	-	1	-	5
1892	1	-	-	-	-	-	1
1893	2	-		-	-	-	2
1894	1	-	-	-	-	-	1
1895	4	-	2	-	-	-	6
1896	1	-	2	-	-	-	4
1897	3	-	-	43	-	-	46
1898	5	-	2	-	1	-	8
1899	10	-	1	-	1	-	12
1900	10	-	1	-	-	-	11
1901	11	-	3	3	1	-	18
1902	45	-	1	-	2	-	48
1903	16	1	2	-	-	-	19
1904	33	2	4	1	-	-	40
1905	61	2	9	-	8	1	81
1906	45	-	1	-	4	1	51
1907	36	-	6	-	4	1	47
1908	20	1	-	-	-	-	21
1909	36	-	1	-	-	-	37
1910	8	-	3	-	-	-	11
1911	74	1	4	-	2	-	81
1912	14	-	1	-	6	-	21
1913	10	-	-	-	1	1	12
1914	4	-	1	-	-	-	5
Totals	495	7	119	50	38	8	717

**Table 2:** Timeline of rabies prophylaxis in Serbia.

Event	Year
Introduction of high taxes for keeping dogs as pets	1834
First official publication on rabies prevention in Serbia	1839
Establishment of a sanitary service for catching stray dogs	1880
Establishment of a comprehensive and permanent record of rabid domestic animals	1883
Foundation of the first Serbian Pasteur Institute in Niš	1900
Foundation of the Pasteur Institute in Novi Sad	1921
Development and introduction of etherized Alivisatos rabies vaccine	1922
Development and introduction of Hempt’s inactivated rabies vaccine	1925
First campaign for the largescale culling of stray dogs	1925–1926
Introduction of post-exposure rabies vaccination of dogs	1926
First prophylactic rabies vaccination of dogs	1935
Introduction of rabies diagnosis by direct immunofluorescence (FAT)	1968
Production of equine rabies immunoglobulin (ERIG) at the Institute of Immunobiology	1970
and Virology Torlak in Belgrade	1980
The last recorded case of human rabies	1980-ties
Replacement of Hempt vaccine by cell culture vaccines for human PEP	1981
Introduction of Flury HEP live attenuated rabies vaccine from chicken embryos for immunization of domestic animals.	1990
Introduction of human rabies immunoglobulin (HRIG) produced at the Blood Transfusion Institute of Serbia in Belgrade Introduction of oral rabies vaccination of foxes and other wild animals	2010

**Table 3:** Bat species sampled for active bat lyssavirus surveillance in Serbia (2007–2008) (from [25]).

Number of Bats Sampled	Bat Species	Ord. No.
3	*Barbastella barbastellus*	1
8	*Eptesicus serotinus*	2
4	*Hypsugo (Pipistrellus) savii*	3
32	*Miniopterus schreibersii*	4
1	*Myotis aurascens*	5
41	*Myotis blythii*	6
33	*Myotis capaccinii*	7
2	*Myotis* cf. *alcathoe*	8
2	*Myotis* cf. *aurascens*	9
44	*Myotis daubentonii*	10
28	*Myotis emarginatus*	11
33	*Myotis myotis*	12
1	*Myotis mystacinus*	13
3	*Myotis nattereri*	14
24	*Nyctalus noctula*	15
15	*Pipistrellus kuhlii*	16
9	*Pipistrellus pipistrellus*	17
3	*Plecotus austriacus*	18
3	*Rhinolophus euryale*	19
22	*Rhinolophus ferrumequinum*	20
311		Total

## References

[1] Mutinelli F., Stankov S., Hristovski M., Seimenis A., Theoharakou H., Vodopija I., King A., Fooks A.R., Aubert M., Wandeler A. (2004). Rabies in Italy, Yugoslavia, Croatia, Bosnia, Slovenia, Macedonia, Albania & Greece. Historical Perspective of Rabies in Europe and the Mediterranean Basin.

[2] Divljanović D. (1974). Infectious Diseases of Domestic Animals in Serbia 1800–1914.

[3] Milanović-Hrašovec I. Medical’s Testimonies of a Writer. https://www.vreme.com/cms/view.php?id=1124020.

[4] Kobliška A. (1896). On canis rabies, diagnosis and prophylaxis. SrpArhCelok Lek.

[5] Nikolić M. (1954). Pasteur’s Institutes in Serbia.

[6] Cabot F. (1899). Report of experimental work on the dilution method of immunization from rabies. J. Exp. Med..

[7] Stevanović M. (1912). Statistics on Antirabies Prophylaxis in Royal Serbian Pasteur Institute for the Last Two Years.

[8] Ivanić S. (1922). Pasteur’s Institutes–Work in 1921.

[9] Alivisatos G. (1922). The protection against Lyssa through the fixed virus. Dtsch. Med. Wochenschr..

[10] Ivanić S. (1924). Zoonoses in Our Country.

[11] Ivanić S., Štampar A. (1926). Epidemiology. Socijalna Medicina.

[12] Milojević V. (1990). Report on the work of the Pasteur Institute in Niš from 1919–1928. Pasteur Institute in Niš 1900–1985.

[13] Hempt A. (1938). On a carbolized anti-rabies aether vaccine and its protective efficacy in man and animal. Behringw. Mitt..

[14] Milovanović V. (1933). Health Cooperatives of Yugoslavia in 1931 and 1932. Med. Yearb. Kingd. Yugosl..

[15] Reports on Rabies of the Pasteur Institute Novi Sad for Years 1953–1991.

[16] Vuković A. (1933). Rabies.

[17] Knežević N., Kosanović P., Lalosevic D. (1996). Rabies vaccine ad us.vet. yesterday, today, tomorrow. Selected Papers of Rabies Prophylaxis.

[18] Serbia Pet Passport & Regulations. https://www.pettravel.com/immigration/Serbia.cfm.

[19] Dean D.J., Abelseth M.K., Atanasiu P., Meslin F.X., Kaplan M.M., Koprowski H. (1996). The fluorescent antibody test. Laboratory Techniques in Rabies.

[20] Koprowski H., Meslin F.X., Kaplan M.M., Koprowski H. (1996). The mouse inoculation test. Laboratory Techniques in Rabies.

[21] Lalošević D. (2001). Spread of Rabies in the South of Europe. Rabies Bull. Eur..

[22] McElhinney L.M., Marston D.A., Freuling C.M., Cragg W., Stankov S., Lalosević D., Lalosević V., Müller T., Fooks A.R. (2011). Molecular diversity and evolutionary history of rabies virus strains circulating in the Balkans. J. Gen. Virol..

[23] Pasteur Institute Novi Sad Rabies Surveillance Information for Animals. https://www.pasterovzavod.rs/en/rabies-surveillance-information-for-animals.

[24] Jelesić Z., Nikolić M. (1956). Isolation of rabies virus from insectivorous bats in Yugoslavia. Bull. World Health Organ..

[25] Vranješ N., Paunović M., Milićević V., Stankov S., Karapandža B., Ungurović U., Lalošević D. Passive and Active Surveillance of Lyssaviruses in Bats in Serbia. Proceedings of the 2nd International Berlin Bat Meeting: Bat Biology and Infectious Diseases.

[26] Brookes S.M., Aegerter J.N., Smith G.C., Derek M., Healy D.M., Jolliffe T.A., Swift S.M., Mackie I.J., Stewart Pritchard J., Racey P.A. (2005). European Bat Lyssavirus in Scottish Bats. Emerg. Infect. Dis..

[27] Hayman D.T.S., Banyard A.C., Wakeley P.R., Harkess G., Marston D., Wood J.L.N., Cunningham A.A., Fooks A.R. (2011). A universal real-time assay for the detection of lyssaviruses. J. Virol. Methods.

[28] Echevarría J.E., Banyard A.C., McElhinney L.M., Fooks A.R. (2019). Current rabies vaccines do not confer protective immunity against divergent lyssaviruses circulating in Europe. Viruses.

[29] Badrane H., Bahloul C., Perrin P., Tordo N. (2001). Evidence of two lyssavirus phylogroups with distinct pathogenicity and immunogenicity. J. Virol..

[30] Banyard A.C., Selden D., Wu G., Thorne L., Jennings D., Marston D., Finke S., Freuling C.M., Müller T., Echevarría J.E. (2018). Isolation, antigenicity and immunogenicity of Lleida bat lyssavirus. J. Gen. Virol..

